# Ustekinumab Drug Survival in Patients with Psoriasis: A retrospective Study of Real Clinical Practice

**DOI:** 10.3390/medicina56110584

**Published:** 2020-10-30

**Authors:** Cristina Galache Osuna, Borja Gómez-Vila, Javier Aubán Pariente, Beatriz Vázquez Losada, Celia Gómez de Castro, Sheila Requena López, Álvaro de Dios Velázquez, Laura Palacios García, Lucía Ordoñez Fernández, Santiago Gómez Diez, Francisco Vázquez López, Jorge Santos-Juanes

**Affiliations:** 1 Department of Dermatology, Hospital Universitario Central de Asturias, 33011 Oviedo, Spain; cristinagalache@gmail.com (C.G.O.); borjagomezvila@gmail.com (B.G.-V.); javiauban@gmail.com (J.A.P.); bvazlosada@gmail.com (B.V.L.); celiagomez_88@hotmail.com (C.G.d.C.); sheilarequenalopez@gmail.com (S.R.L.); aldedivel@gmail.com (Á.d.D.V.); llaurinapalacios@hotmail.com (L.P.G.); santi15gomez@gmail.com (S.G.D.); fvazquezlopez2@gmail.com (F.V.L.); 2 Clinical Management Unit, UGC Farmacia, Hospital Universitario Central de Asturias, 33011 Oviedo, Asturias, Spain; lucia.ordonez@sespa.es; 3 Area of Dermatology, Department of Medicine, University of Oviedo, 33011 Oviedo, Asturias, Spain; 4 Instittudo de Investigadión Sanitaria del Principado de Asturia (ISPA), Instituto Universitario Oncológico del Principado de Asturias (IUOPA), 33011 Oviedo, Asturias, Spain

**Keywords:** psoriasis, biological treatment, ustekinumab, interleukin drug survival

## Abstract

*Background and objectives:* The efficacy and safety of ustekinumab have been proved in clinical trials. In daily clinical practice, knowing the factors that determine survival differences of biological drugs allows psoriasis treatment to be optimized as a function of patient characteristics. The main objectives of this work are to understand ustekinumab drug survival in patients diagnosed with plaque psoriasis in the Hospital Universitario Central de Asturias (HUCA Dermatology Department, and to identify the predictors of drug discontinuation. *Materials and Methods*: A retrospective hospital-based study, including data from 148 patients who were receiving ustekinumab (Stelara^®^) between 1 February 2009 and 30 November 2019, were collected. Survival curves were approximated through the Kaplan–Meier estimator and compared using the log-rank test. Proportional hazard Cox regression models were used for multivariate analyses while both unadjusted and adjusted hazard ratios (HR) were used for summarizing the studied differences. *Results:* The average duration of the treatment before discontinuation was 47.57 months (SD 32.63 months; median 41 months). The retention rates were 82% (2 years), 66% (5 years), and 58% (8 years). Median survival was 80 months (95% confidence interval. CI 36.9 to 123.01 months). The survival study revealed statistically significant differences between patients with arthritis (log-rank test, *p* < 0.001) and those who had previously received biological treatment (log-rank test, *p* = 0.026). The five-year prevalence in patients still under treatment was 80% (those without arthritis) and 54% (arthritis patients). In the multivariate analysis, only the patients with arthritis had a lower rate of drug survival. No statistically significant differences were observed for any of the other comorbidities studied. The first and second most frequent causes of discontinuation were secondary failure and arthritis inefficacy, respectively. *Conclusion:* Ustekinumab is a biological drug conferring high survival in plaque psoriasis patients. Ustekinumab survival is lower in patients with arthritis.

## 1. Introduction

Psoriasis [1] is a chronic inflammatory disease of the skin. Its etiology is multifactorial, in which the genetic susceptibility of an individual interacts with environmental factors to produce dysregulation of the immune system [2].

It is characterized by a greater level of proliferation of keratinocytes and by the infiltration of immunocompetent cells in the epidermis and dermis. It affects approximately 2% of the world’s population. At present, it is considered a systemic disease with predominantly cutaneous manifestations and is associated with psoriatic arthritis in up to 30% of cases [3]. It has two periods of highest incidence, from 20 to 30 years, and from 50 to 60 years [4].

Psoriasis exhibits variable morphology, distribution, extension and clinical course, and may be classified into one of several forms: plaque (vulgar), guttate, erythrodermic, generalized pustular psoriasis, and palmoplantar pustulosis. Plaque psoriasis is the most frequent clinical form, accounting for 90% of all patients with psoriasis [5]. The diagnosis is clinical and, in most cases, does not need histological confirmation. It manifests with the presence of erythematous lesions of variable size with well-defined borders, covered with silvery scales. It usually occurs symmetrically, in any region of the body surface, but most frequently on the knees, elbows, scalp and trunk [3]. Based on the location and extent of the lesions, it may be classified as mild, moderate, or severe.

The etiology of psoriasis is not fully understood, but it is thought to be a product of the interaction between genetic, immune, and environmental factors. From 30 to 50% of patients with psoriasis report having affected first- or second-degree relatives [6]. The most consistently related locus is PSORS1, which is located on chromosome 6 [7,8].

*Psors1* involves, among other genes, HLA-Cw6 * 0602, which is related to a more aggressive psoriasis course, earlier onset, greater extent of skin involvement, poorer response to treatments and a greater frequency of comorbidities [7,8].

Of the immunological factors currently studied, the interleukin (IL)-23/Th17 axis is considered to be crucial to the pathogenesis of psoriasis. Indeed, IL-23 is produced mainly by antigen-presenting cells, and induces the differentiation of Th17 and Th22 cells, which produce pro-inflammatory cytokines such as IL-17A, IL-17F, IL-22, and IL-26, which mediate the epidermal hyperplasia, keratinocyte activation, and tissue inflammation inherent to psoriasis [9,10].

Stress is the best-known influential environmental factor. Szepietowski reported 60% of patients with psoriasis as experiencing stressful circumstances. Infections, trauma, certain drugs, and even cold weather and seasonal changes are other frequent triggers [11].

An epidemiological association of psoriasis has been reported with diseases whose shared chronic inflammation is a common pathogenic substrate. Associated comorbidities or diseases usually manifest years after the onset of the disease and appear more frequently in patients with severe psoriasis [12]. It has been linked to psoriatic arthritis, Crohn’s disease, metabolic syndrome and all its components, liver steatosis, pulmonary involvement, depression, bipolar disorder, anxiety, addictions such as alcoholism and smoking, tumors such as lymphomas and solid tumors, and, more recently, to changes in renal function [13,14].

It is also associated with a greater risk of mortality from cardiovascular events and a variety of other causes [12,14].

Psoriatic arthritis is present in 5 to 30% of these patients, being most prevalent in those with more severe cutaneous psoriasis. It is a chronic inflammatory disease of the musculoskeletal system that is also mediated by the immune system, and affects both sexes equally, usually occurring after skin involvement [15]. Ustekinumab (Stelara^®^) is a human IgG1k monoclonal antibody, approved by the FDA in 2009 for the treatment of moderate–severe plaque psoriasis. By binding to the p40 protein subunit of human IL-12 and IL-23 these cytokines are unable to bind to their receptor protein, IL-12Rβ1, which is expressed on the surface of immune cells [16]. In this way, differentiation and clonal expansion of the TH1 and TH17 lymphocyte subpopulations are inhibited. The decrease in these and their cytokines resolves the inflammation that occurs in the psoriasis plaques [17].

The survival of a drug is defined as the duration of a specific therapy, the period over which a certain drug remains a suitable option for a specific patient [18].

Some authors interpret drug survival as being a marker of therapeutic success, since it depends on efficacy, the presence of adverse reactions, and the patients’ satisfaction with the treatment [17].

Another aspect to consider is the time that the patient has to continue without the drug for the treatment to be considered to have been suspended. This period varies from drug to drug, but, in the case of ustekinumab, it is considered to be 24 weeks without receiving the drug (the time equivalent to two doses) [18].

Analyzing the survival of biological therapy for psoriasis can inform us about the efficacy of drugs over time in real clinical practice, in which doses are adjusted depending on how the disease evolves and adjuvant treatments are administered, and patient satisfaction is a key factor influencing the introduction of therapeutic changes. None of these actions is allowed in clinical trials. In clinical practice, this type of analysis can be influenced by changes in the prices of biological treatments, the appearance of new drugs, and changes in prescription habits [19]. For these reasons, great caution is recommended when comparing the results of survival studies of different drugs [20]. In addition, there are few real-life data evaluating ustekinumab in patients with psoriasis particularly over the long-term.

The objectives of this study were to determine survival of the drug ustekinumab and to study the factors that predict discontinuation in patients diagnosed with plaque psoriasis in the HUCA Dermatology Department.

## 2. Materials and Methods

We designed a retrospective, hospital study, with an inclusion period between 1 February 2009 and 30 November 2019. The study was approved by the Ethics and Research Committee of the Principality of Asturias, Spain (Number 113-18).

The study included 148 patients who received 45 or 90 mg ustekinumab (Stelara) for the treatment of plaque psoriasis from the HUCA Dermatology Department. We only included patients starting Ustekinumab due to dermatological indication.

The records of all patients who received this treatment were reviewed. The following data were obtained from each patient’s clinical history: sex, age (years), weight (kilograms), height (centimeters), family history of psoriasis (a positive family history was considered if there was psoriasis in at least one first degree relative), age of onset of skin pathology, whether the patient had received previous treatments (“non-naive”) or not (“naive”), and presence of arthritis (diagnosed by a rheumatologist). Comorbidities such as high blood pressure, diabetes mellitus (DM), and dyslipidemia were identified. The medications taken were reviewed, coding the patients as having hypertension, DM, or dyslipidemia if they declared it, if it was evident from their clinical history, or if they were taking antihypertensive, antidiabetic, or lipid-lowering medication. Patients with a BP of >135/85 mm Hg, measured during their consultation, were also classified as hypertensive.

The analytical determinations in the medical records were reviewed. Patients with hypertriglyceridemia (triglycerides > 150 mg/dL), hypercholesterolemia (total cholesterol > 200 mg/dL), or low-density hyperlipidemia (LDL > 160 mg/dL) were considered to have dyslipidemia.

Body mass index (BMI) was calculated as weight (kg)/square of height (m). Following World Health Organization (WHO) guidelines, values of BMI ≥ 30 were taken to indicate obesity. Descriptive data of the patient cohort are summarized as total frequencies and percentages.

Ustekinumab survival (retention rate) was retrospectively calculated as the time period until definitive treatment interruption after initiation treatment.

### Statistical Analysis

Statistical analyses were performed with IBM SPSS version 24.0 (IBM, Armonk, NY, USA). Data are presented as mean ± standard deviation for continuous variables, and number and percentage for categorical variables. 

The chi-square test was used for qualitative variables. Survival curves were approximated through the Kaplan-Meier estimator and compared using the long-rank test. Proportional hazard Cox regression models were used for multivariate analyses while both unadjusted and adjusted hazard ratios (HR) were used for summarizing the studied differences. 95% confidence intervals (95% CI) are also provided. The proportionality of the risks was previously checked through the Schoenfeld residual.

We selected the following variables as possible predictors: sex, age of onset of psoriasis, family history, obesity, arthritis, previous use of biologics, arterial hypertension, and dyslipidemia. Group differences were considered to be statistically significant for values of *p* < 0.05. 

Final sample size allows to declare significative (Type I error of 0.05), with a probability of 0.2 (Type II error), those hazard ratios above 1.75, difference in proportions above 25% and standardized differences of means above 0.5.

## 3. Results

### 3.1. Patient Characteristics 

The study included 148 patients, all of whom were of white ethnicity. Their characteristics are summarized in Table 1. It is worth highlighting that there were twice as many men as women in the sample, a predominance of psoriasis patients with a family history, early onset, and a considerable number with concomitant arthritis.

### 3.2. Drug Survival

**Figure 1:**
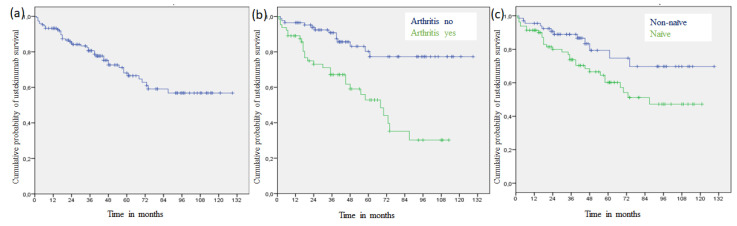
(**a**). Kaplan–Meier curve of ustekinumab survival. (**b**) Kaplan–Meier curves of ustekinumab survival according to arthritis (*p* < 0.001). (**c**) Kaplan–Meier curves of ustekinumab survival according to naivety status (*p* = 0.026).

The survival of the drug is shown in Figure 1a.

In our cohort, the retention rates were 82% (2 years), 66% (5 years), and 58% (8 years), and the median survival was 80 months (95% confidence interval, CI 36.90 to 123.01 months). Log-rank tests revealed no significant differences in drug survival in relation to the presence of diabetes (*p* = 0.296), arterial hypertension (*p* = 0.578), dyslipidemia (*p* = 0.258), family history of member with psoriasis (*p* = 0.175), age of onset of psoriasis after 40 years of age (*p* = 0.501), obesity (*p* = 0.110), or sex (*p* = 0.453).

However, there were significant differences with respect to the presence of arthritis (*p* < 0.001) (Figure 1b) and having previously had a biologic treatment (*p* = 0.026) (Figure 1c). The probability of remaining on the treatment after 5 years of follow-up was significantly higher in patients without than with arthritis (80% vs. 54%; *p* < 0.001) (Table 2).

### 3.3. Univariate and Multivariate Analysis

Univariate analyses showed statistically significant differences between groups with respect to the presence of arthritis and the previous use of a biologic (Table 3). The probability that a patient would discontinue treatment was 3.6 times higher among those with than those without arthritis and the naive patients were at 2.1-fold lower risk to discontinuous treatment that the non-naive patients.

In the multivariate model, only arthritis retained its statistical significance. Previous use of biologics was not independently significant in the model.

### 3.4. Patients Who Discontinue Treatment 

At the end of the study, 104 of the 148 patients in the study (70.27%) were still pursuing their treatment. There were a variety of reasons why the other 44 (29.73%) patients did not continue: primary failure (lack of initial efficacy, prior to 16 weeks of treatment) (6 patients; 5.77%), secondary failure (loss of efficacy over time, superior to 16 weeks of treatment) (17, 11.49%), infections (1 each with upper respiratory tract infection and urinary tract infection; 0.96%), women wishing to become pregnant (2, 1.92%), paradoxical pustular reaction (1, 0.96%), death (2, 1.92%), hepatocarcinoma (1, 0.96%), lost to follow-up (1, 0.96%), headache and edema (1, 0.96%), dizziness (1, 0.96%) and psoriatic arthritis inefficacy (10, 9.62%) (Table 4). Exitus and the development of hepatocarcinoma were causes of suspension but could not be attributed to ustekinumab. No differences between men and women were found in terms of primary or secondary failure or adverse effects.

Table 5 shows the strategies employed before changing ustekinumab in patients with secondary therapeutic failure, lack of joint activity, and patients who continued with their intensified treatment. Only two patients in the series were de-intensified every 16 weeks.

Table 5 shows the strategies employed before changing ustekinumab in patients (n) with secondary therapeutic failure, arthritis inefficacy, and patients who continued with their intensified treatment. Only two patients in the series were de-intensified every 16 weeks.

## 4. Discussion

We present a retrospective study in which we assess the overall survival of the drug ustekinumab, based on data collected over 10 years and 10 months in the HUCA Dermatology Department.

Several studies of the survival of ustekinumab in the treatment of psoriasis have been published: the PSOLAR [21], BADBIR [22], DERMBIO [23], BIOCAPTURE [24], and SNIIRAM [25] studies, and the Hungarian NHIF database [26], all of which are characterized by a short follow-up time. In addition, there are hospital-based studies such as those of Galluzzo et al. [27], Kishimoto et al. [28], and the eight-year-long study by Elberdin et al. [29]. Two meta-analyses have also been carried out of the survival of the various biologics used to treat psoriasis [30,31].

The demographic characteristics of our patient cohort are comparable to those of the BIOBADADERM [18], PSOLAR [21], BADBIR [22], DERMBIO [23], the Hungarian NHIF database [26], and the Elberdin et al. [29] studies in terms of the proportions of the sexes of the patients (a predominance of males; 66%), and the age of initiation of treatment with ustekinumab.

Regarding the overall survival of the drug, retention rates of 92% (first year), 82% (second year), 66% (fifth year), and 58% (eighth year) were noted. Subsequently, the rate was stable until 12 years. One-year survival in our study was towards the higher end of the range of the other studies, being surpassed only by that of the PSOLAR registry [21], and similar to those of previous studies of national databases, such as the BADBIR [22] and DERMBIO [23] studies. Ustekinumab survival was higher in our study than in the ORBIT and BIOBADADERM studies, whose conditions most closely resembled ours [18,32] In studies with a longer follow-up time, survival at eight years was similar to that noted in our cohort [27]. Ustekinumab yields the longest survival of all currently known biologics [33]. The meta-analysis published in Scientific Reports [30] highlighted the excellent short-term efficacy of ustekinumab, but noted the decrease in survival over time, from 82% in the first year to 56% after four years of treatment. In our study, the probability of continuing with the drug for four years was 72%, reducing to 58% by eight years.

Knowing the factors that influence survival can be key for choosing the most appropriate treatment with the available biological drugs. Our current knowledge indicates that obesity, being female, and having previous had a biologic treatment are factors that reduce the survival of biological drugs [23,30,31].

In our series, we did not find that obesity or female sex influenced survival with ustekinumab. This is consistent with the findings of the BADBIR [22] and ORBIT [32] studies but differs from those of the DERMBIO [23] and BIOCAPTURE [24] studies, in which obese patients and women had poorer survival. The latter results are the opposite of that of Galluzzo et al., who found women to have longer survival [27]. We found statistically significant differences between naive and non-naive patients from the Kaplan–Meier and univariate analyses, but these effects proved not to be independently significant when considered in the multivariate study. This is a similar finding to those of several studies [24,27,31], but different from the results of the PSOLAR [21], BADBIR [22], DERMBIO [23], and BIOCAPTURE [24] studies, in which naive patients were found to have better survival than non-naive patients.

Our multivariate analysis suggests that the most influential factor is the presence of arthritis, and it can be deduced that these patients receive more treatments with biologics, similar to the conclusions of other studies [25].

Our series features more patients with DM, arterial hypertension, dyslipidemia, and psoriatic arthritis than those of the BIOBADADERM [18], BADBIR [22], GALLUZO [27], and ELDERDIN [29] studies. We attribute these differences to the active search for these patients undertaken by the HUCA psoriasis clinic, which has already been widely reported in the literature [34,35,36,37].

These comorbidities have not been found to be related to higher or lower rates of survival of biological therapy in any of the series [31], except for the BADBIR study, which attributed a notably worse survival in patients with DM to the greater drug resistance arising from a higher level of continuous inflammation [22].

42.8% of our patients had arthritis, a lower figure than reported in our review of patients treated with secukinumab (53.1%) [38] and with adalimumab (56.7%) [39]. This ranged from 9.8% in the Galluzzo et al. series [27] to 33.6% in the SNIIRAM series [25].

Unlike the present study, in which we found worse survival in patients with arthritis than in those without (96% vs. 89% in the first year; 91% vs. 67% in the third year), survival was independent of whether patients had arthritis in the DERMBIO [23], BIOCAPTURE [23], and ORBIT [32] studies, and in the Italian and Elderdin et al. series [27,29]. This is likely to have been due to the small number of patients with arthritis in those series, which meant that a real relationship of a broadly similar magnitude to the one we noted would not have been statistically significant with such a small sample size (beta-type error). It has recently been reported that retention rate of ustekinumab 90 mg was 76.1% after a median time of 12 months follow up in patients with psoriatic arthritis in a real-world setting [40]. Studies investigating ustekinumab survival on a large cohort of patients with psoriatic arthritis, in order to replicate these findings, are required. 

In the BADBIR study [22], the presence of arthritis was associated with a 42% relative risk of suspending ustekinumab treatment, which is quite similar to our own estimate of 34%. In that study, 15.5% of the patients had arthritis, but the figure was based on a large sample of 3118 patients.

The main cause of drug discontinuation is secondary failure (38.64% of cases of discontinuation), followed by arthritis inefficacy (22.73%), and adverse effects (11.36%). Except for our findings on arthritis inefficacy, which is barely mentioned in the literature, our results are similar to those of the series that considered causes of discontinuation [18,23,30,31]. As the exception, the Elderdin et al. study found that 6.25% of patients who suspended did so because of a lack of joint activity [29]. The safety of the drug is demonstrated by the fact that only 5 of the 148 patients (3.38%) suspended treatment due to adverse effects, like the levels previously reported [1,23,30,31]. 

Regarding the differences noted between the pivotal studies and those carried out under clinical conditions, it is important to point out that different strategies were employed for 27 patients in our series before suspending treatment that are not permissible in clinical trials and that may have helped increase the median survival of the drug. With this drug we were able to deintensify only two patients due to good disease control, increasing the interval between doses from 12 to 16 weeks. This contrasts with adalimumab, with which we were able to deintensify 63.5% of patients, producing a good response [39].

### Limitations of the Study

Our study has several limitations. First, it is observational in nature, and so is prone to the effects of biases inherent to this type of study. Second, the selection of treatments for each patient is not randomized in routine clinical practice. Third, new active psoriasis treatments have been introduced that are able to modify drug survival. Fourth, the published studies, two theoretical meta-analyses of national registries (to which only some hospitals contribute data) and hospital studies similar to ours, used different statistical approaches and examined different variables, which makes it impossible to compare those results directly with ours.

## 5. Conclusions

Ustekinumab drug survival in our sample was towards the highest end of the range of all the equivalent published series (92% in the first year; 58% in the eighth year).

Of the variables studied, sex, age, early onset of psoriasis, family history of psoriasis, dyslipidemia, diabetes mellitus, arterial hypertension, and previous use of biologics were unrelated to survival; only the presence of arthritis was associated with significantly lower survival of ustekinumab, bestowing a relative risk of 34% of suspending treatment for affected patients compared with those who did not suffer from arthritis. This information will help patients make an informed decision when starting a biologic therapy, based on drug survival outcome.

## Figures and Tables

**Table 1:** Baseline characteristics of patients.

**Sex (male), n (%)**	99 (66.9%)
**Age at start of biologic treatment (years), mean ± SD**	47.97 ± 13.43
**Positive family history of psoriasis (yes), n (%)**	97 (65.5%)
**Onset before 40 years of age (%)**	118 (79.7%)
**Duration of treatment (months); mean ± SD; median**	47.57 ± 32.63; 41
**Comorbidities, n (%)**	
**Obesity (BMI ≥ 30)**	57 (38.5%)
**Diabetes mellitus**	32 (21.6%)
**Arterial hypertension**	50 (33.8%)
**Dyslipidemia**	87 (58.8%)
**Arthritis**	64 (43.2%)
**Prior treatments with biologics (%)**	81 (54.7%)
**One biologic**	53
**Two biologics**	17
**Three biologics**	9
**Four biologics**	2

**Table 2:** Cumulative probability of ustekinumab survival, and according to arthritis status at different time intervals.

Percentage (95% CI)	1 year	2 years	3 years	4 years	5 years	8 years
**Global**	92 (88–96)	82 (76–88)	75 (69–81)	68 (60–76)	66 (56–76)	58 (46–68)
**Arthritis: yes**	89 (81–97)	75 (63–87)	67 (55–78)	62 (48–76)	54 (40–68)	31 (15–47)
**Arthritis: no**	96 (92–100)	94 (88–100)	91 (85-97)	85 (75–95)	80 (68–92)	77 (69–85)

**Table 3:** Cox regression analyses. Hazard ratios for risk of ustekinumab discontinuation.

Univariate Analyses	*p*	HR (95% CI)
**Psoriasis onset ≥ 40 years**	0.514	0.785 (0.386–1.597)
**Sex (male)**	0.456	0.789 (0.423–1.471)
**Obesity: BMI ≥ 30**	0.115	0.614 (0.335–1.127)
***Arthritis: yes***	<0.001	3.623 (1.876–6.993)
**Diabetes: yes**	0.301	0.702 (0.359–1.372)
**Hypertension arterial: yes**	0.580	0.839 (0.450–1.564)
**Dyslipidemia: yes**	0.263	1.443 (0.759–2.741)
**Family history: yes**	0.181	0.624 (0.313–1.244)
***Naive patients:***	0.03	0.476 (0.243–0.932)
**Multivariate analysis**	*p*	95% CI
***Arthritis: yes***	0.001	3.344 (1.639–6.849)
**Naive patients**	0.286	0.683 (0.339–1.736)

**Table 4:** Patients who discontinued and reasons for discontinuation. Others: death (2), hepatocarcinoma (1), pregnancy desire (2), loss to follow-up (1). (Chi-square test).

Ustekinumab (Total)/Discontinuations n (%)	148 (100%)/44 (29.72%)	Men (99)	Women (49)	*p*
Discontinuations	44 (100%)	28 (100%)	16 (100%)	0.024
Primary failure	6 (13.64%)	5 (17.85%)	1 (6.25%)	0.230
Secondary failure	17 (38.64%)	12 (42.85%)	5 (31.25%)	0.868
Arthritis inefficacy	10 (22.73%)	5 (17.85%)	5 (31.25%)	0.082
Adverse events	5 (11.36%)	3 (10.71%)	2 (12.50%)	0.747
Others	6 (13.64%)	3 (10.71%)	3 (18.75%)	0.391

**Table 5:** Strategies before changing ustekinumab.

	Secondary Failure	Arthritis Inefficacy	Patients Intensified
**Methotrexate added (n) (dose)**	3 (15 mg/week)	3 (15 mg/week)	1 (15 mg/week)
**Period between treatments shortened (n) (dose)**	2 (90 mg/8 weeks) 2 (90 mg/10 weeks)	2 (90 mg/8 weeks)	
**Dose increased from 45 mg to 90 mg**	1	5	8
